# Protein kinase D enzymes are dispensable for proliferation, survival and antigen receptor-regulated NFκB activity in vertebrate B-cells

**DOI:** 10.1016/j.febslet.2007.02.055

**Published:** 2007-04-03

**Authors:** Ping Liu, Andrew M. Scharenberg, Doreen A. Cantrell, Sharon A. Matthews

**Affiliations:** a Division of Cell Biology and Immunology, College of Life Sciences, University of Dundee, Dow Street, Dundee, DD1 5EH, Scotland, UK; b Department of Paediatrics and Immunology/Children’s Hospital and Regional Medical Centre, University of Washington, USA

**Keywords:** PKD, protein kinase D, PKC, protein kinase C, BCR, B cell antigen receptor, Protein kinase D, PKD, Proliferation, Survival, NFκB, HSP27

## Abstract

To investigate the importance of protein kinase D (PKD) enzymes we generated a PKD-null DT40 B-lymphocyte cell line. Previously we have shown that PKDs have an essential role in regulating class II histone deacetylases in DT40 B-cells [Matthews, S.A., Liu, P., Spitaler, M., Olson, E.N., McKinsey, T.A., Cantrell, D.A. and Scharenberg, A.M. (2006) Essential role for protein kinase D family kinases in the regulation of class II histone deacetylases in B lymphocytes. Mol. Cell Biol. 26, 1569–1577]. We now show that PKDs are also required to regulate HSP27 phosphorylation in DT40 B-cells. However, in contrast to previous observations in other cell types, PKD enzymes do not regulate basic cellular processes such as proliferation or survival responses, nor NFκB transcriptional activity downstream of the B cell antigen receptor. Thus, PKDs have a selective role in DT40 B-cell biology.

## 1 Introduction

The protein kinase D (PKD) serine/threonine kinase family has three members: PKD1, PKD2 and PKD3. Most cell types express at least two PKD isoforms but PKD enzymes are especially highly expressed in haematopoietic cells, where they are activated in response to antigen receptors stimulation [2,3]. A conserved signalling pathway linking antigen receptors to PKDs involves the activation of PLCγ and the subsequent production of diacylglycerol (DAG) which stimulates classical and/or novel protein kinase Cs (PKC) that phosphorylate two key regulatory serine residues in the activation loop of PKD kinases [3–6]. The N-terminal regulatory region of PKD enzymes contains a DAG binding domain and direct binding of DAG also contributes to PKD1 activation [7] as well as regulating the spatial location of PKD enzymes within cells [8–12].

PKD enzymes have been proposed to regulate numerous cellular functions, including cell proliferation [13–16], anti-apoptotic signals [17,18] and thymocyte development [19]. Expression of mutant catalytically inactive and constitutively activated PKDs can also modify Golgi function, cell adhesion and cell motility (reviewed in [20]). In particular, PKDs have been widely linked to the activation of the NFκB transcription factor and in regulating cell survival during oxidative stress [17,21–23]. Another recently proposed PKD1 substrate is HSP27 [24], a small heat shock protein involved in regulating cell migration and cell survival [25]. An essential role for PKD enzymes in regulating class II histone deacetylases (HDACs), enzymes that repress MEF2-dependent gene transcription, has also been demonstrated [1,26–28].

To investigate the biological role of PKDs we have generated DT40 B cell lines that lack expression of one or more members of the PKD family [1], allowing us to investigate the function(s) of PKD isoforms following B cell antigen receptor (BCR) stimulation, as well addressing the issue of functional redundancy between the different PKD family members. Previous studies have shown that PKDs are indispensable for HDAC regulation in B cells [1]. Herein we show that PKDs are also indispensable for HSP27 phosphorylation in B cells. However, PKD-null DT40 B cells are viable and proliferate normally. Moreover, loss of the entire cellular pool of PKD does not critically affect oxidative stress responses in B cells nor do PKD kinases play an essential role in regulating NFκB transcriptional activity. Together, these findings reveal that in B lymphocytes, PKD kinases are not critical regulators of many of the cellular processes previously ascribed to them in other cellular systems.

## 2 Materials and methods

### 2.1 Cell culture, transient transfections and cell stimulation

The generation, culture and activation of PKD1^−/−^, PKD3^−/−^ and PKD1/3^−/−^ knockout DT40 B cell lines have been described previously [1]. Cells were lysed and protein extracts were analysed in Western blotting experiments as previously described [1]. Chloramphenicol acetyl transferase assays have been described previously [29].

### 2.2 sIgM staining

DT40 B cells (2 × 10^6^ cells per point) were resuspended in 200 μl buffer (RPMI 1640 media, 1% foetal calf serum) containing anti-chicken M1 monoclonal antibody conjugated to FITC for 20 min on ice. The cells were washed twice and fluorescent intensity was analysed by flow cytometry.

All results shown are representative of at two to four independent experiments unless otherwise indicated.

## 3 Results

### 3.1 Loss of HSP27 phosphorylation in DT40 B cells lacking expression of PKD family kinases

**Fig. 1:**
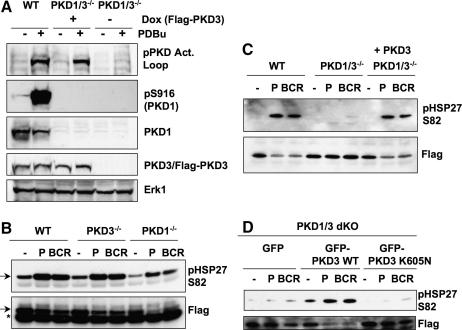
(A) Expression and activation of PKD enzymes in wild-type and PKD1/3^−/−^DT40 B cells. Cells were treated ± = 25 ng/ml PdBu for 10 min and analyzed by Western blotting of whole cell extracts with the indicated antibodies. PKD1/3^−/−^DT40 B cells were either continuously maintained in doxycycline (to maintain Flag-PKD3 expression) or were washed out of doxycycline for 5 days (to generate a PKD-null phenotype). (B–D) HSP27 phosphorylation in PKD1/3^−/−^ DT40 B cells. Wild-type, PKD1^−/−^, PKD3^−/−^ and PKD1/3^−/−^ DT40 B cells were transiently transfected with a Flag-HSP27 expression construct. In Fig. 1D, PKD1/3^−/−^ DT40 B cells were co-transfected with Flag-HSP27 and either GFP, GFP-PKD3 wild-type or GFP-PKD3 K605 N constructs, as indicated. The cells were then left untreated or were stimulated with either 10 μg/ml M 4(BCR) or 25 ng/ml PdBu( P) for 30 min. Flag immunoprecipitates were analysed by SDS–PAGE and Western blotting the indicated antibodies. ∗ = IgL chain.

DT40 B cells express two PKD isoforms, PKD1 and PKD3, and as previously described we have recently generated DT40 B cell lines that lack expression of either PKD1 or PKD3 or both enzymes [1]. In generating the double knockout cell lines we targeted the PKD1 loci in a PKD3^−/−^ cell line that expressed a Flag-PKD3 transgene under the control of a doxycycline-inducible promoter. Hence, in the presence of doxycycline, Flag-PKD3 expression in PKD1/3 double knockout cells is comparable to endogenous PKD3 present in wild-type DT40 cells and removal of doxycycline from the culture media for 5 days results in a completely null PKD phenotype (Fig. 1A).

Previously, we have demonstrated that phosphorylation and nuclear exclusion of class II histone deacetylases (HDACs) during BCR engagement is defective in PKD1/3^−/−^ B cells and can restored upon re-expression of a single PKD isoform [1]. The small heat shock protein HSP27 has recently been proposed as a PKD1 substrate [24] and we accordingly assessed whether PKD-null DT40 cells have defective phosphorylation of HSP27 on serine 82, the proposed PKD1 substrate sequence. We initially investigated the regulation of HSP27 phosphorylation in single knockout DT40 B cells lacking either PKD1 or PKD3. As shown in Fig. 1B, activation of the BCR or treatment with the DAG-mimetic PdBu increased the levels of HSP27 phosphorylation at S82 in wild-type DT40 B cells. BCR and phorbol ester signals were also able to increase HSP27 phosphorylation in PKD1 or PKD3 single knockout DT40 B cells (Fig. 1B). However, BCR- and phorbol ester-induced phosphorylation of HSP27 on S82 was abolished in B cells that lacked both PKD1 and PKD3 (Fig. 1C). Significantly, doxycycline-induced expression of the Flag-PKD3 transgene in the double knockout cells was sufficient to restore normal regulation of HSP27 phosphorylation (Fig. 1C). In contrast, expression of a kinase-deficient PKD3 mutant protein in the double knockout cells was not able to restore BCR- or phorbol ester-induced HSP27 phosphorylation (Fig. 1D). Hence, PKD3 as well as PKD1 can regulate HSP27 phosphorylation and in DT40 B cells they are functionally redundant as HSP27 kinases.

### 3.2 Cellular proliferation and survival in DT40 B cells lacking expression of PKD family kinases

**Fig. 2:**
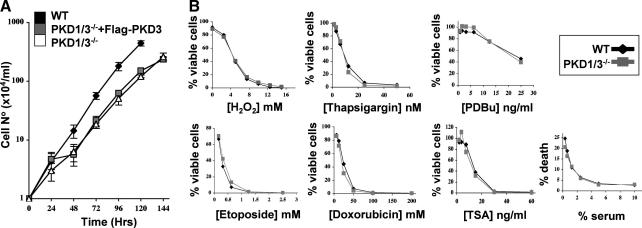
Growth and cell survival responses in PKD1/3^−/−^ DT40 B cells. (A) Exponentially growing wild-type, PKD1/3^−/−^/PKD3^+^ and PKD1^−/−^3^−/−^ (washed out of doxycycline for at least 5 days) DT40 B cells were plated at a cell density of 1 × 10^4^/ml and cell number was counted daily. Results shown are the mean ± S.E. of triplicate cultures and are representative of two to three independent experiments. (B) Wild-type and PKD1/3^−/−^ DT40 B cells were cultured at a density of 1 × 10^5^ cells/ml with increasing concentrations of the indicated stimuli. After 24–48 h in culture, the number of viable cells in the cultures was determined by flow cytometry, using CalBRITE (Becton Dickinson) beads for quantitation of cell numbers. The data are expressed as a percentage of the number of viable cells present in control, non-treated cultures for each cell line (untreated = 100%). In some cases, the data are presented as the % cell death after 24 h as determined by flow cytometry, gating on 7AAD^+^ cells. All results are representative of at least two to four independent experiments. TSA, Trichostatin A.

PKD enzymes have previously been linked to the regulation of cell proliferation and survival (reviewed in [20]). To investigate the effect that loss of PKD kinases had on B cell survival and/or proliferation we cultured wild-type and PKD-null cells in the presence (PKD1/3^−/−^: Flag-PKD3^+ve^) or absence (PKD1/3^−/−^) of doxycycline and monitored exponential growth. As shown in Fig. 2A, PKD1/3^−/−^ cells proliferated exponentially and re-expression of Flag-PKD3 in these cells had no impact on the rate of proliferation. Furthermore, the viability of PKD1/3^−/−^ B cells during routine culturing was not significantly different from that of wild-type B cells (data not shown). It was noted that the population doubling time of PKD1/3^−/−^ cells was slightly slower than that of wild type DT40 cells (12.7 ± 2.8 h versus 10.2 ± 0.4 h) but the failure of PKD3 re-expression to modify the proliferation rate of PKD1/3^−/−^ cells suggests that these small differences were most likely the result of clonal variation and were not caused specifically by loss of PKD enzymes. Thus, PKD family enzymes are not essential for regulating basal survival and proliferation of DT40 B cells.

PKD enzymes, specifically PKD1 and PKD2, have previously been linked to a protective role against oxidative stress-induced injury in 3T3 fibroblast, HeLa and epithelial cell lines [17,30–32]. We therefore addressed the role of PKD family kinases in regulating B cell survival in response to oxidative stress and other stress stimuli. As shown in Fig. 2B, loss of PKD1/3 expression had no significant impact on the survival of DT40 B cells in response to mitochondrial stress stimuli (H_2_O_2_ or serum deprivation); DNA damaging agents (etoposide or doxorubicin); ER pathway stress due to calcium overload (thapsigargin) or following prolonged treatment with phorbol esters or Trichostatin A, an inhibitor of class I/II HDACs. Thus, PKD kinases do not play an essential role in regulating B cell survival in response to a range of different stress stimuli.

### 3.3 Antigen receptor regulated signalling pathways in PKD-null DT40 B cells

**Fig. 3:**
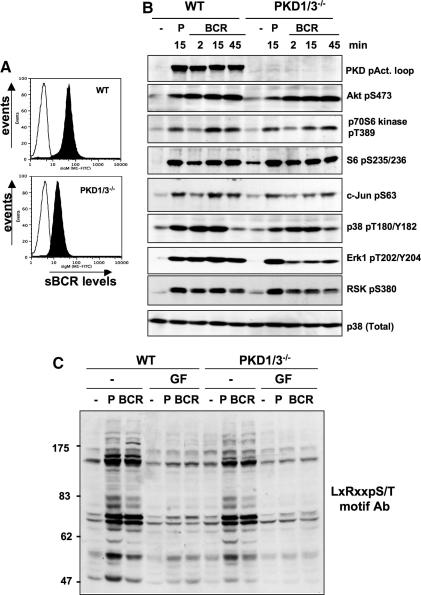
BCR-induced signalling events in PKD1/3^−/−^ DT40 B cells. (A) Flow cytometric analysis of surface BCR expression (as detected by IgM levels) in wild-type, PKD1/3^−/−^/PKD3^+^ and PKD1/3^−/−^ DT40 cells. (B) Wild-type and PKD1/3^−/−^ DT40 cells were cultured in 0.5% FBS-containing media for 6 h, then with or without 10 μg/ml M4 (BCR) or 25 ng/ml PdBu (P) for the indicated times and cellular extracts were analysed by SDS–PAGE and Western blotting with the indicated antibodies. (C) Wild-type and PKD1/3^−/−^ DT40 cells, pretreated for 1 h with 5 μM GF109203X (GF) or DMSO solvent (−) were stimulated with or without 25 ng/ml PdBu (P) or 10 μg/ml M4 (BCR) for 30 min before cellular extracts were analysed by SDS–PAGE and Western blotting with a phospho-LxRxxpS/T motif antibody (CST, #4381).

To further explore the contribution of PKD kinases to DT40 B cell biology we investigated whether specific BCR-regulated signalling events were defective in the PKD-null B cells. Initial experiments revealed that surface expression of the BCR was reduced in PKD1/3^−/−^ (and in PKD1/3^−/−^:Flag-PKD3^+ve^) cells compared to wild-type DT40 B cells (Fig. 3A and data not shown). Nevertheless, BCR-crosslinking of PKD1/3^−/−^ cells was sufficient to induce the activation of a number of signalling cascades, similar to that observed in wild-type cells (Fig. 3B). Hence, BCR-induced activation of the Akt, mTOR/p70 S6 kinase (as shown by S6 ribosomal protein phosphorylation) and MAPK signalling pathways was clearly detectable in PKD1/3-null B cells (Fig. 3B). Furthermore, enhanced tyrosine phosphorylation of multiple cellular proteins as well as an increase in intracellular calcium levels was also observed following BCR stimulation of PKD1/3-null B cells (data not shown). We did observe that the strength of BCR (but not phorbol ester)-induced regulation of the Erk1-RSK1 signalling pathway was reduced in PKD1/3^−/−^ B cells compared to wild-type B cells (Fig. 3B). One interpretation of this data is that PKD enzymes may modulate Erk activation. Indeed, PKD enzymes have previously been linked to the growth factor-regulated Erk signalling in fibroblast and endothelial cell lines [33–35]. However, BCR-induced Erk phosphorylation was also reduced in PKD1/3^−/−^-Flag-PKD3^+^ B cells (data not shown) suggesting that reduced BCR levels on the surface of PKD1/3^−/−^ (and PKD1/3^−/−^-Flag-PKD3^+^) B cells may itself impact on the strength of activation of this specific intracellular signalling pathway.

To search for other potential PKD targets that may show defective regulation in PKD1/3^−/−^ DT40 B cells, we used a PKD substrate phospho-antibody that recognises consensus phosphorylation sequences targeted by PKD enzymes (LxRxxpS/T) [36]. As shown in Fig. 3C, phorbol ester- and BCR-induced phosphorylation of cellular substrates detected by this phospho-antibody was similar in wild-type and PKD1/3^−/−^ cells and is therefore independent of PKD enzymes. However, pretreatment of both wild-type and PKD1/3^−/−^ DT40 B cells with GF109203X, a bisindoylmaleimide derivative that inhibits PKCs prevented the induction of proteins that contain phosphorylated LxRxxS/T motifs. Thus loss of PKD1/3 enzymes does not globally disrupt the phosphorylation of cellular proteins that contain LxRxxpS/T motifs. This result is perhaps not surprising as LxRxxS/T motifs also act as good substrates for other serine/threonine kinases such as MAPKAPK2. However these experiments do provide further evidence that phosphospecific antisera are not sufficiently selective to be designated kinase specific substrate antisera.

BCR-induced signalling pathways culminate in the activation of gene transcription events that control B cell survival, proliferation and function. In this context, it has been proposed that PKD family members control of gene transcription through activation of the NFκB transcription factor. Thus, PKD-mediated activation of NFκB occurs downstream of a variety of different signals, including mROS/oxidative stress, lysophosphatidic acid and the Bcr-Abl oncogene [17,21,23,30,37]. Furthermore, expression of an activated PKD1 mutant enhances HPK1-mediated NFκB activation [38]. In B cells, NFκB is known to be regulated via DAG and PKCβ [39,40] but whether PKDs are key intermediaries for NFκB regulation has not been explored.

**Fig. 4:**
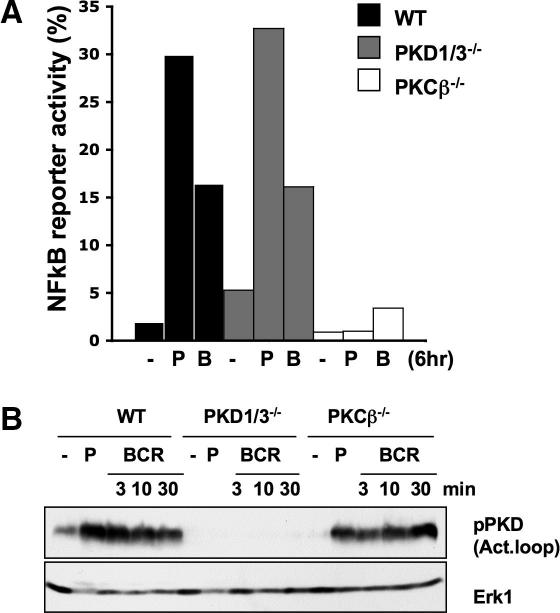
NFκB transcriptional activity in PKD1/3^−/−^ DT40 B cells. (A) The indicated DT40 B cell lines were transiently transfected with an NFκB-CAT reporter construct. The cells were then left untreated or were stimulated with either 10 μg/ml M4 (B) or 25 ng/ml PdBu (P) for 6 h before the activity of the reporter construct was analysed as described in Section 2. (B) Expression and activation of PKD enzymes in wild-type, PKD1/3^−/−^ and PKCβ^−/−^ DT40 B cells was performed as described in Fig. 1A.

The data (Fig. 4A) show that NFκB transcriptional activity was strongly induced in both wild-type and PKD1/3^−/−^ DT40 B cells in response to either phorbol ester or BCR stimulation. In contrast, BCR and phorbol ester-induced NFκB transcriptional activity was abolished in PKCβ^−/−^ DT40 B cells (Fig. 4A), although strong activation of PKD kinases (as assessed by autophosphorylation of PKD1 at S916) was observed in the PKCβ^−/−^ cells (Fig. 4B). Thus, PKD kinases are neither essential nor sufficient to mediate BCR-induced NFκB activation in DT40 B cells and hence do not participate in DAG/PKC mediated control of NFκB.

## 4 Discussion

Protein kinase D serine kinases have been proposed to regulate diverse cellular functions including the phosphorylation and nuclear localisation of class II HDACs and the phosphorylation of HSP27. It has also been suggested that PKDs act as mitochondrial sensors for oxidative stress and play a role in regulating NFκB transcription factors [41]. Most of the data about the function of PKDs has come from experiments that ectopically express active or inhibitory PKD mutants or that use RNAi to reduce PKD expression. We have used gene targeting to specifically delete PKD alleles in DT40 chicken B cells and can thus use PKD-null DT40 cells to assess the relative contribution of individual PKD isoforms in class II HDAC control versus oxidative stress responses and NFκB regulation in lymphocytes. We have previously used these PKD-null DT40 cells to define an essential role for PKDs in regulation of class II HDACs, the present report now describes an indispensable role for PKDs in regulating the phosphorylation of HSP27 on serine 82, a site previously identified as a target for the p38-MAPKAPK2 signalling cascade [42]. However, studies of PKD-null DT40 cells reveal that PKD family kinases are not essential for oxidative stress survival responses nor are they required for activation of NFκB transcription factors. These latter findings are in striking contrast to previous observations in HeLa and epithelial cell lines where overexpression/RNAi approaches have implicated PKD1/2 in the control of proliferation, survival and NFκB activation [20,23]. Hence, the present report shows that the proposed roles for PKDs as key sensors that modulate survival pathways in response to oxidative stress and regulate cell survival and proliferation are not ubiquitous and may be restricted to certain cell lineages. Taken together, these data indicate that loss of expression of PKD family members does not globally impact on early BCR-regulated signalling pathways.
